# Preparation of a Carbon-Based Solid Acid Catalyst by Sulfonating Activated Carbon in a Chemical Reduction Process

**DOI:** 10.3390/molecules15107188

**Published:** 2010-10-18

**Authors:** Xiao-Yan Liu, Miao Huang, Hai-Long Ma, Zeng-Qiang Zhang, Jin-Ming Gao, Yu-Lei Zhu, Xiao-Jin Han, Xiang-Yun Guo

**Affiliations:** 1 College of Science, Northwest A&F University, Yangling 712100, Shaanxi, China; 2 State Key Laboratory of Coal Conversion, Institute of Coal Chemistry, CAS, Taiyuan 030001, China

**Keywords:** sulfonate, activated carbon, solid acid, catalysts

## Abstract

Sulfonated (SO_3_H-bearing) activated carbon (AC-SO_3_H) was synthesized by an aryl diazonium salt reduction process. The obtained material had a SO_3_H density of 0.64 mmol·g^−1^ and a specific surface area of 602 m^2^·g^−1^. The catalytic properties of AC-SO_3_H were compared with that of two commercial solid acid catalysts, Nafion NR50 and Amberlyst-15. In a 10-h esterification reaction of acetic acid with ethanol, the acid conversion with AC-SO_3_H (78%) was lower than that of Amberlyst-15 (86%), which could be attributed to the fact that the SO_3_H density of the sulfonated carbon was lower than that of Amberlyst-15 (4.60 mmol·g^−1^). However, AC-SO_3_H exhibited comparable and even much higher catalytic activities than the commercial catalysts in the esterification of aliphatic acids with longer carbon chains such as hexanoic acid and decanoic acid, which may be due to the large specific surface area and mesoporous structures of the activated carbon. The disadvantage of AC-SO_3_H is the leaching of SO_3_H group during the reactions.

## 1. Introduction

Sulfonated (SO_3_H-bearing) carbon materials have been reported to act as strong solid acid catalysts. Hara’s group first prepared sulfonated carbon catalysts via the sulfonation and carbonization of polycyclic aromatic hydrocarbons [1]. These catalysts showed excellent activity in a series of acid-catalyzed reactions, although, they were not stable enough and the aromatic molecules leached out above 100 ºC [2]. The problem could be overcome by selecting saccharides as carbon precursor [2,3,4,5,6,7]. The controlled carbonization and sulfonation of these materials resulted in stable carbon structures with a high SO_3_H group density. Carbon-based solid acid catalysts could also be obtained by sulfonation of carbon nanotubes [8] or mesoporous carbon materials [9,10,11]. 

Activated carbon (AC) is widely used as a catalyst support in a variety of industrial and environmental applications for its chemical stability, high specific surface area, and low cost [12]. Studies on the sulfonation of AC are however quite limited [13,14,15]. An early experimental result from Hara’s group showed that heating AC in H_2_SO_4_ only produced a carbon material with a very low SO_3_H group density (less than 0.15 mmol·g^−1^), a fact attributed to the chemical inertness of AC [1]. Onda *et al*. reported the generation of a sulfonated carbon material with a SO_3_H density of 0.44 mmol·g^−1^ by treatment of AC with concentrated H_2_SO_4_ [13,14]. The obtained carbon material in this case was quite stable and showed evident catalytic activity for the hydrolysis of cellulose. Recently, another paper from Hara’s group described the preparation of a porous sulfonated carbon catalyst with high specific surface area by carbonization and sulfonation of ZnCl_2_-impregnated wood powders [15].

Besides the H_2_SO_4_ treatment method, chemical reduction of aryl diazonium salts has also been proved to be quite efficient for the functionalization of carbon materials [16]. Feng *et al*. reported the preparation of carbon-based solid acid catalysts by covalent attachment of SO_3_H radicals on the surface of ordered mesoporous carbon in a chemical reduction process [17,18]. However, so far there have been no reports on sulfonation of AC using the chemical reduction method. Here we report the functionalization of AC with SO_3_H-containing group by reacting AC with 4-benzenediazonium sulfonate using hypophosphorous acid (H_3_PO_2_) as reducing agent. The structure and catalytic properties of the obtained material were also examined.

## 2. Results and Discussion

### 2.1. Material characteristics

**Figure 1:**
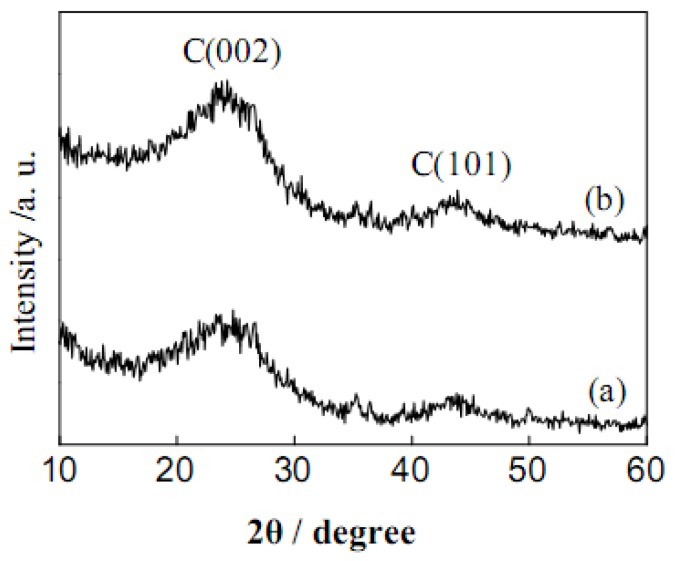
XRD patterns for activated carbon (a) before and (b) after sulfonation.

Figure 1 shows the XRD patterns of AC and the sulfonated carbon material (AC-SO_3_H). The broad C (002) diffraction peak (2θ = 15-30º) can be attributed to the amorphous carbon structures. The weak and broad C (101) diffraction peak (2θ = 40-50º) is due to the *a* axis of the graphite structure [1,2,3,4,5,6]. There is no noticeable difference in the XRD patterns between AC and AC-SO_3_H, suggesting that the chemical reduction process does not affect the microstructure of carbon materials.

The SO_3_H density of sulfonated carbon material was determined by combining the S elemental analysis and the total acid site density results. As shown in Table 1, the S contents in AC before and after sulfonation were 0.26 and 0.90 mmol·g^−1^, respectively. The total acid density of AC increased from 0.30 to 1.01 mmol·g^−1^ via the sulfonation.

**Figure 2:**
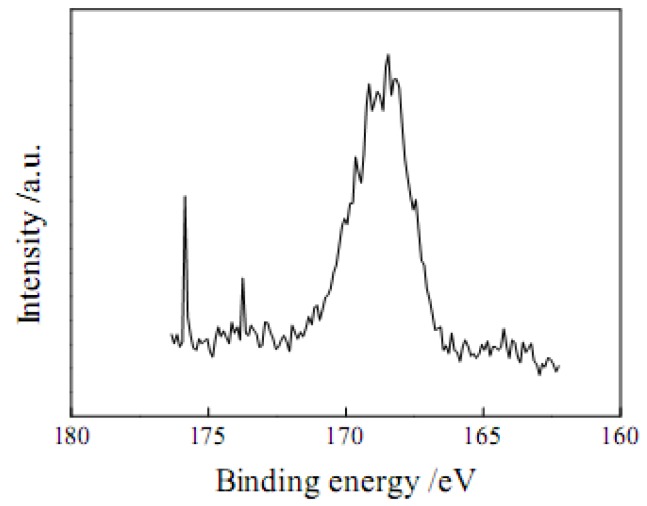
S 2p XPS spectrum of AC-SO_3_H.

These results showed that the increment of S content (0.64 mmol·g^−1^) was close to that of the total acid density (0.71 mmol·g^−1^) in the sulfonation process. Therefore the SO_3_H density of sulfonated AC is about 0.64 mmol·g^−1^. X-ray photoelectron spectroscopy (XPS) analysis was conducted to further verify the chemical state of sulfur in AC-SO_3_H. As shown in Figure 2, a strong S 2p peak appears at about 168 eV, which can be denoted as SO_3_H group [3]. 

**Figure 3:**
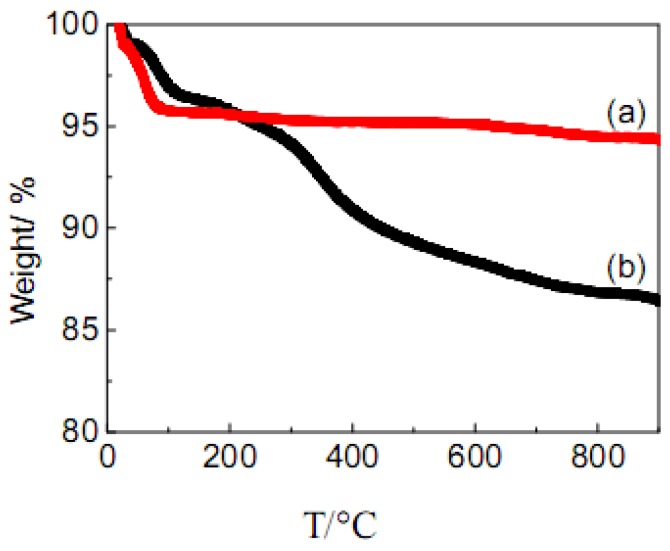
Thermogravimetric profiles for (a) AC, (b) AC-SO_3_H.

The thermal stability of AC-SO_3_H was studied by TGA results (under flowing N_2_). As shown in Figure 3, AC showed a slight weight loss (about 5%) below 150 ºC, which was mainly due to the loss of a small amount of adsorbed water. The weight loss process of AC-SO_3_H below 150 ºC was similar to that of AC. However, the thermogravimetric curve of AC-SO_3_H at higher temperatures showed a more significant weight loss compared to that of AC, which is mainly due to the gradual desorption and thermal decomposition process of PhSO_3_H group.

**Figure 4:**
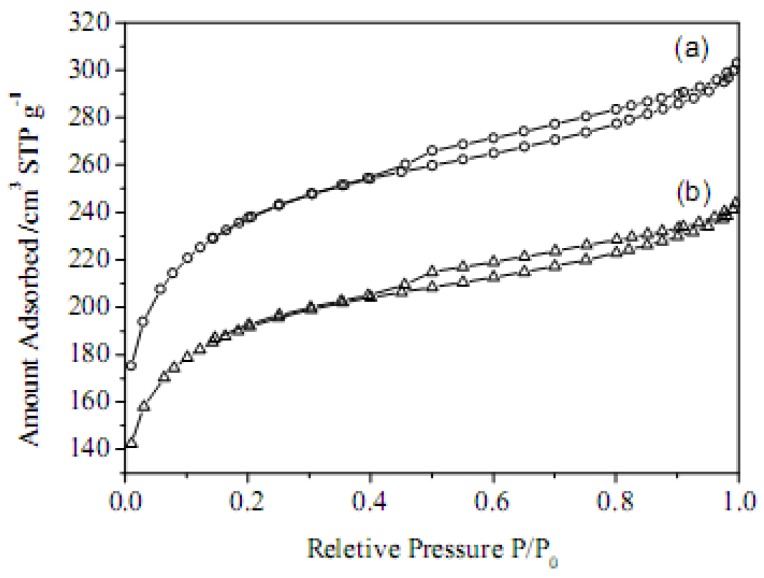
N_2_ adsorption isotherms of (a) AC and (b) AC-SO_3_H.

Figure 4 shows the N_2_ adsorption isotherms of the carbon materials. Both AC and AC-SO_3_H exhibit similar isotherms with evident hysteresis loops in the relative pressure range of about 0.4-0.9. The surface properties of the carbon materials are summarized in Table 1. After sulfonation, the surface area and pore volume of the carbon materials decrease from 751 m^2^·g^−1^ and 0.47 cm^3^·g^−1^ to 602 m^2^·g^−1^ and 0.38 cm^3^·g^−1^, suggesting that a small part of the pore space has been occupied by the grafted SO_3_H groups. The pore diameter does not change during sulfonation (from 2.5 to 2.4 nm).

### 2.2. Catalytic properties

**Figure 5:**
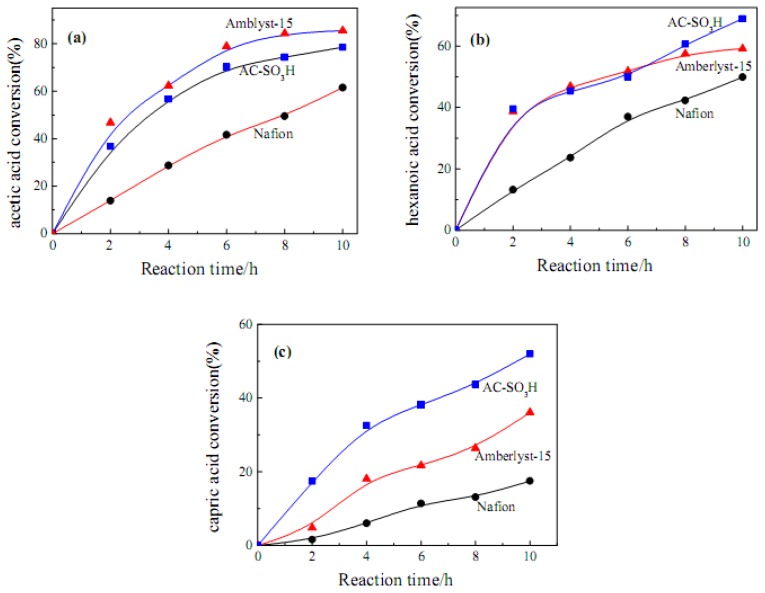
Acid catalyzed esterification of several aliphatic acids with ethanol: (a) acetic acid, (b) hexanoic acid, and (c) decanoic acid.

The catalytic activity of AC-SO_3_H has been investigated in the esterification of three kinds of aliphatic acids with different carbon chain lengths (acetic, hexanoic and decanoic acid), as shown in Figure 5. The results for two commercial solid acid catalysts such as Amberlyst-15 and protonated Nafion NR50 are also shown for comparison. From Figure 5(a), the highest acetic acid conversion (86%) is obtained with Amberlyst-15 after 10 h of reaction. AC-SO_3_H gives an acid conversion of 78%. The conversion with Nafion is 61%. As shown in Table 1, the SO_3_H density of Amberlyst-15 (4.60 mmol·g^−1^) is much higher than that of AC-SO_3_H (0.64 mmol·g^−1^) and Nafion (0.45 mmol·g^−1^). Therefore, it may be conferred that the catalytic activity for acetic acid esterification is mainly dependent on the acid density of the catalyst. 

Figure 5(b) shows the esterification results of hexanoic acid with ethanol. The catalytic activity of AC-SO_3_H is comparable in this case with that of Amberlyst-15. The advantage of our carbon catalyst becomes more evident in the esterification of decanoic acid, as shown in Figure 5(c). The decanoic acid conversion with AC-SO_3_H (52%) is much higher than that with Amberlyst-15 (36%) and with Nafion (17%) after 10 h of reaction. The results in Figure 5(b) and Figure 5(c) suggest that the catalytic activity for esterification of large aliphatic acid molecules is not solely dependent on the acid density. The surface properties of catalysts such as specific surface area and pore diameters are also quite important. As shown in Table 1, AC-SO_3_H has a large surface area (602 m^2^·g^−1^) and high density of mesopores, which may be more convenient for larger aliphatic acid molecules to adsorb on the catalyst surface and approach to the active acid sites. Therefore, our sulfonated carbon materials may have potential applications in the acid-catalyzed reactions of large organic molecules. 

To investigate the stability of SO_3_H group on the carbon catalyst, AC-SO_3_H was reused in a series of acetic acid esterification reaction cycles. Before each cycle, AC-SO_3_H was washed with 1 mol·L^−1^ H_2_SO_4_, and then washed repeatedly with deionized water at least seven times until no SO_4_^2−^ ions were detected in the washed water [11].

**Figure 6:**
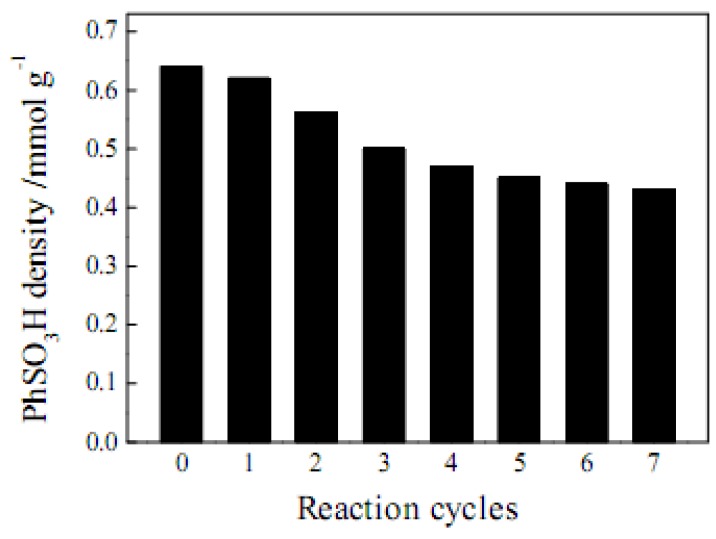
Variation of the SO_3_H density on the carbon catalysts in a series of 2-h acetic acid esterification reaction cycles.

Figure 6 shows the variation of SO_3_H density in the reaction cycles. The fresh catalyst has a SO_3_H density of 0.64 mmol·g^−1^. The value decreases evidently in the beginning four reaction cycles and then remains stable at 0.42 mmol·g^−1^ in the following three cycles. The results indicate that about 34% SO_3_H group (0.22 mmol·g^−1^) of the fresh catalyst will leach out during the reactions, while 64% SO_3_H is preserved. It has been pointed out elsewhere that the leaching of SO_3_H groups is a common problem for sulfonated catalysts [7]. More efforts should be made in the future to enhance the stability of SO_3_H groups.

## 3. Experimental Section

### 3.1. Materials and Reagents

Activated carbon was purchased from the Taiyuan Xinhua Activated Carbon Company (Taiyuan, Shanxi Province, P.R. China). Commercial solid acid catalysts, Amberlyst-15 and protonated Nafion NR50, were purchased from Alfa Aesar (Tianjin, China). All other reagents were in reagent grade and used without any further treatment. 

### 3.2. Catalyst preparation

The sulfonated carbon material was obtained by the reaction of 4-benzene-diazonium sulfonate with activated carbon using hypophosphorous acid (H_3_PO_2_) as the reducing agent [17,18]. 4-Benzene-diazonium sulfonate was synthesized by diazotization of 4-aminobenzenesulfonic acid [17]. In a typical process, 4-aminobenzenesulfonic acid (13.0 g) was dispersed in 1M HCl (75 mL) in a three-necked flask. An ice-water bath was used to control the temperature at 0-5 ºC. Then NaNO_2_ (83 mL, 1 M) was added dropwise into the flask. The mixture was stirred for 50 min. The obtained white precipitate was filtered and washed with cold water. 

To functionalize AC, distilled water (100 mL), ethanol (100 mL), diazonium salt (12.0 g) and AC (2.0 g) were added in a three-necked flask. Then 50 wt % H_3_PO_2_ (100 mL) was added and the temperature was controlled at 0-5 ºC. After stirring for 30 min, another portion of 50 wt % H_3_PO_2_ (100 mL) was added and the mixture was stirred for another 30 min. The sulfonated AC is filtered and washed with distilled water at least seven times and then with acetone, and dried at 80 ºC. 

### 3.3. Catalyst characterization

The sulfur content of carbon materials and commercial catalysts (Amberlyst-15 and Nafion NR50) was determined by a Sulfur Analyzer (KZDL-4) [19]. The total acid site density of the samples was obtained using acid-base back titration [7]. Typically, the sample (0.1 g) was mixed with NaOH aqueous solution (50 mL, 0.004 M) while stirring for 1 h at room temperature. Then excess NaOH was neutralized with HCl (0.02 M). The obtained carbon material was characterized by XRD (Rigaku D/MAX-2400), XPS (Thermo Scientific K-Alpha), N_2_ adsorption analysis (Micromeritics ASAP300), and TGA (Netzsch STA 449C).

### 3.4. Catalytic reactions

The catalytic properties of the sulfonated carbon material were examined through the esterification of a series of aliphatic acids (acetic acid, hexanoic acid, and decanoic acid) with ethanol at 70 ºC. Typically, aliphatic acid (0.1 mol), ethanol (1.0 mol), and the carbon catalyst (or the commercial catalysts) (0.2 g) were used in the reactions. The liquid reaction mixtures were analyzed by gas chromatography (Agilent GC 7890). 

## 4. Conclusions

A porous carbon-based solid acid catalyst possessing a SO_3_H density of 0.64 mmol·g^−1^ and a specific surface area of 602 m^2^·g^−1^ was synthesized by sulfonating activated carbon in an aryl diazonium salt reduction process. In the esterification reaction of acetic acid with ethanol, AC-SO_3_H showed inferior catalytic activity to the commercial catalyst, Amberlyst-15, because the former’s SO_3_H density is much lower than the latter’s (4.60 mmol·g^−1^). However, AC-SO_3_H possessed evident advantages in the esterification of decanoic acid, which was probably due to the high specific surface area and a large number of mesopores of the activated carbon. Our study results suggest that AC-SO_3_H may have potential applications in the acid-catalyzed reactions of large organic molecules. The main disadvantage of AC-SO_3_H is the leaching of SO_3_H groups during the reactions. 

## Figures and Tables

**Table 1:** The textural properties of the materials.

Samples	N_2_ adsorption^(a)^			S content (mmol g^−1^)	Total acid density (mmol H^+^g^−1^)	SO_3_H density (mmol g^−1^)
	S. A.	P. V.	P. D.			
AC	751	0.47	2.5	0.26	0.30	0
AC-SO_3_H	602	0.38	2.4	0.90	1.01	0.64
Nafion NR50	<1			0.45	0.49	0.45
Amberlyst-15	45			4.60	4.66	4.60

^(a)^ BET surface area (S. A.) in m^2^ g^−1^, pore volume (P. V.) in cm^3^ g^−1^, pore diameter (P. D.) in nm.
